# Bovine Transcription Factor POU Class 2 Homeobox 1 (POU2F1/Oct1) Protein Promotes BoHV-1 Replication in MDBK Cells

**DOI:** 10.3390/v16101549

**Published:** 2024-09-30

**Authors:** Enguang Rong, Inga Dry, Robert G. Dalziel, Wenfang Spring Tan

**Affiliations:** The Roslin Institute and Royal (Dick) School of Veterinary Studies, University of Edinburgh, Easter Bush Campus, Midlothian EH25 9RG, UKinga.dry@roslin.ed.ac.uk (I.D.);

**Keywords:** BoHV-1, viral transcription, Oct1, CRISPR/Cas9

## Abstract

Bovine herpesvirus type 1 (BoHV-1) causes severe diseases in bovine species and great economic burden to the cattle industry worldwide. Due to its complex life cycle, many host factors that affect BoHV-1 replication remain to be explored. To understand the possible roles that the Oct1 cellular protein could play in this process, we first created Oct1-deficient MDBK cells using CRISPR/Cas9-mediated genome editing. Upon infection, the absence of Oct1 in MDBK cells significantly impacted BoHV-1 replication, a phenotype rescued by over-expressing the wild-type Oct1 protein in the deficient cells. We further found that the expression of all three classes of temporal genes, including essential and non-essential viral genes, were significantly reduced in Oct1 knockout MDBK cells, following both high and low multiplicity of infection. In summary, our findings confirm that the bovine Oct1 protein acts as a pro-viral factor for BoHV-1 replication by promoting its viral gene transcription in MDBK cells.

## 1. Introduction

As a major animal health threat, bovine herpesvirus 1 (BoHV-1) infection leads to significant economic losses across the worldwide cattle industry [1,2,3]. BoHV-1 infection can cause various health problems in dairy and beef cattle, such as infectious bovine rhinotracheitis (IBR), infectious pustular vulvovaginitis, balanopostitis, and abortions [2,4]. In addition, it significantly reduces milk yields in dairy cows [5]. BoHV-1 has wide presence around the world. Within Europe, two recent serological surveys have suggested that IBR was endemic in the UK, and that the majority of UK herds (around 80%) were infected with BoHV-1 [6]. Similar serological surveys of Irish dairy and beef herds estimated the herd seroprevalence to be between 21% and 70% [7,8]. The BoHV-1 infection status of animals, semen, and embryos could restrict their international trade in EU countries [9].

As a member of the alphaherpesvirus subfamily, acute infection by BoHV-1 leads to cellular apoptosis, inflammatory responses, and shedding of infectious progenies. After clearance of the primary infection, the virus becomes dormant in sensory neurons, and stressful life events often trigger reactivation from latency throughout the lifetime of the animal. During active replication, BoHV-1 employs various host mechanisms to facilitate self-propagation, a process analogous to those of other alphaherpesviruses. Briefly, upon cell entry and docking at the nuclear pore, the capsid injects its viral genome into the nucleus to unleash three waves of viral gene expression: immediate early (IE), early (E), and late (L) [4]. Three IE proteins, IPC0, IPC4, and ICP27, along with the virion protein VP16, are some of the major regulators of alphaherpesvirus gene transcription [10]. Besides these genes, Circ [11,12], as well as UL21, UL33, and UL34, have been indicated by high throughput sequencing [12] as having expression dynamics of an IE gene, although the IE status of UL21, UL33, and UL34 has been disputed in a separate study [11]. Among these, ICP4 is the main regulator and recruits cellular transcription factors to viral promoters to enhance transcription initiation, a process essential for efficient lytic infection [10].

Out of the many cellular transcription factors, the transcription factor POU class 2 homeobox 1 (POU2F1/Oct1) is a versatile regulator of both host and viral genes that contain the TAATGARAT motif [13,14]. Although ubiquitously expressed, it has been shown to regulate both ubiquitous and tissue specific cellular genes [15,16]. In addition to cellular genes, Oct1 protein is also required for efficient transcription of genes from multiple viruses such as the human hepatitis B virus [17,18], mouse mammary tumor virus [19], Epstein–Barr virus [20], human herpes simplex virus (HSV-1) [21], and potentially the BoHV-1 virus [22,23]. During an active infection of HSV-1, Oct1 forms a complex with viral factor VP16 and another host cell factor HCF-1 capable of binding to TAATGARAT motifs [22,23] of IE promoters and induces viral gene transcription [24,25,26]. Besides promoting viral gene transcription, Oct1 has also been shown to repress virus-induced interferon alpha (IFN-α) gene expression [27]. These studies suggest that Oct1 is a critical transcriptional regulatory component for several viral systems; however, its proposed role within the context of a BoHV-1 infection has not been confirmed by direct experimental evidence.

In this manuscript, we describe a series of experiments conducted to dissect the role Oct1 plays during active BoHV-1 infection using reverse genetics. To achieve this, we first generated Oct1 knockout (KO) MDBK cells using CRISPR/Cas9 gene targeting. These cells were then challenged with BoHV-1 at high and low MOI to examine virus growth kinetics. We also conducted qPCR experiments to determine whether transcription efficiency of viral genes in these KO cells were affected.

## 2. Materials and Methods

### 2.1. Cell Culture

Madin-Darby bovine kidney (MDBK) cells were maintained in Dulbecco’s modified Eagle’s medium (DMEM) supplemented with 5% heat inactivated horse serum (HS), 0.1 mM nonessential amino acids, 1 mM sodium pyruvate, 2 mM L-glutamine and 1% (vol/vol) penicillin-streptomycin solution in an atmosphere of 5% CO_2_ at 37 °C. All cell culture reagents were purchased from Gibco/Invitrogen (Carlsbad, CA, USA) or Merck (Darmstadt, Germany).

### 2.2. Establishing Oct1-Deficient MDBK Cells

sgRNAs targeting Oct1 were synthesized by in vitro transcription as described in Tan et al. [28], using the primers listed in Table 1 for generating the DNA templates. After purification and quality control, 1 ug of each sgRNA was electroporated into 500,000 Cas9 positive MDBK cells [28] using a Neon Electroporator and the 100 µL tip (1200 v, 30 ms, and two pulses). After dilution cloning and colony picking, ~500bp amplicons encompassing the region targeted by the sgRNAs were obtained by PCR with gene-specific primers F1 + R1 (Table 1) and sequenced by Sanger. Clones with indels in the Oct1 gene were selected, expanded, and further characterized.

### 2.3. Establishment of MDBK Cells over Expressing Bovine Oct1

The coding region of bovine Oct1 (XM_005203424.4) was amplified from the cDNA of wild-type MDBK cells using the primers in Table S1 and cloned into a piggyBac expression plasmid with a His-tag and a Neomycin selection marker [29]. Oct1 −/− (clone A7) or WT cells were co-electroporated with the hyperactive piggyBac transposase (pCMV-hypBase) [30] and the cDNA expression plasmid, empty vector (EV), or PBS using the Neon Electroporator as above. After two days of recovery, the transfected cells were then selected with neomycin (400 μg/mL). Single cell clones were picked up and expanded to establish as stable clonal cell lines. The ectopic Oct1 gene expression in these monoclonal cell lines was examined by western blotting.

### 2.4. Western Blotting

MDBK cells selected with Neomycin from above were harvested and lysed with the IP lysis buffer (Abcam, Cambridge, MA, USA). Afterwards, Laemmli sample buffer with DTT (Bio-Rad, Hercules, CA, USA) was added, and the samples were boiled for 5 min at 95 °C. These samples were then resolved on a 12% SDS-PAGE gel and blotted onto a polyvinylidene difluoride membrane (Immobilon-P, Millipore, Billerica, MA, USA). After blocking and 1 h incubation with an anti-His or anti α-Tubulin (1:1000; Sigma, St. Louis, MO, USA) primary antibody, a secondary horseradish peroxide-conjugated antibody (1:5000) was added to the membrane for conjugation. The blots were then visualized using the enhanced chemiluminescence method (ECL, Thermo Fisher, Carlsbad, CA, USA), followed by exposure with the BioMax X-ray film.

### 2.5. Viral Infections

A modified BoHV-1 virus based on the Cooper strain was used in this study, with GFP fused to its small capsid protein VP26 [31]. For experiments with the alcelaphine gammaherpesvirus 1 (AlHV-1) virus, the wild-type strain C500 was used [32]. All viral stocks were propagated in wild-type MDBK cells as described previously [33]. The BoHV-1 and AlHV-1 viruses were maintained in a biosecurity level 2+ laboratory approved by the University of Edinburgh. For the time course experiment, Oct1 knockout cells along with WT cells were inoculated with the BoHV-1 virus at an MOI of 0.01, 0.1, 1, or 10. Total viral samples were harvested at 0, 2, 4, 6, 8, 10, 12, 16, 18, 24, 36, 48, or 72 hpi and titrated on WT MDBKs. Total mRNA samples were also obtained to determine the dynamics of viral gene expression.

### 2.6. Plaque Assays

Plaque assays were carried out as previously described [28]. Briefly, 4~6 × 10^5^ cells were seeded in six-well plates, and the next day, monolayers of MDBK cells were incubated with serial diluted BoHV-1 virus samples for 1 h at 37 °C with intermittent rocking. The inoculums were then removed, and the cells were washed with phosphate-buffered saline (PBS). The cells were then overlaid with DMEM containing 2% HS and 0.5% Avicel microcrystalline cellulose (FMC BioPolymer, Philadelphia, PA, USA). At day 4 after infection, the monolayers were fixed and stained with toluidine blue dye solution (0.1% toluidine blue O, 20% methanol). Finally, all six-well plaque plates were scanned and analyzed by ImageJ software (version 1.52). The total areas of random selected plaques were measured and normalized to the averages obtained from WT cells (set to 1).

### 2.7. Growth Rate Assessment of KO Cell Lines

Non-KO WT cells (Clone P), −/− cells (clones A7, 2A2, N19) and +/− cells (clone 2A5) were plated on a 96-well tissue culture plate at 1000 cells per well and incubated for five days. The cell numbers were then estimated using the CellTiter-Glo 2.0 Cell Viability Assay by following manufacturer instructions.

### 2.8. Semiquantitative RT-PCR

Total RNA from the various Oct1-deficient MDBK cells was isolated using the RNeasy Plus Mini Kit (Qiagen, Hilden, Germany). The RNA was then DNase-treated and the cDNA was synthesized using a QuantiTect reverse transcription kit (Qiagen, Hilden, Germany). The mRNA levels of Oct1 transcript variants and the housekeeping gene GAPDH were then detected by qPCR using the primers listed in Table S2 with a cycle number chosen while the PCR was still in the log phase. The resulting PCR products were resolved on electrophoresis on 2% agarose gels and photographed under UV light.

### 2.9. Quantitative RT-PCR

To determine viral gene transcription dynamics, cDNA samples from infected MDBK cells were prepared as above. qPCR was carried out using the LightCycler 480 System and LC480 SYBR Green 1 Master (Roche) to quantify the transcript levels of 21 viral genes using the primers list in Table S3. qPCR was also conducted on 18S rRNA to serve as internal controls and to normalize template input. Gene differential expression between samples was calculated using the 2-ΔΔCT method. As there were no CT values for BoHV-1 gene-specific primers in mock-infected samples, the values from the 24 hpi CTRL samples at MOI of 10 were used as the normalization factors.

## 3. Results

### 3.1. Oct1 Knockout in MDBK Cells Using CRISPR/Cas9

**Figure 1:**
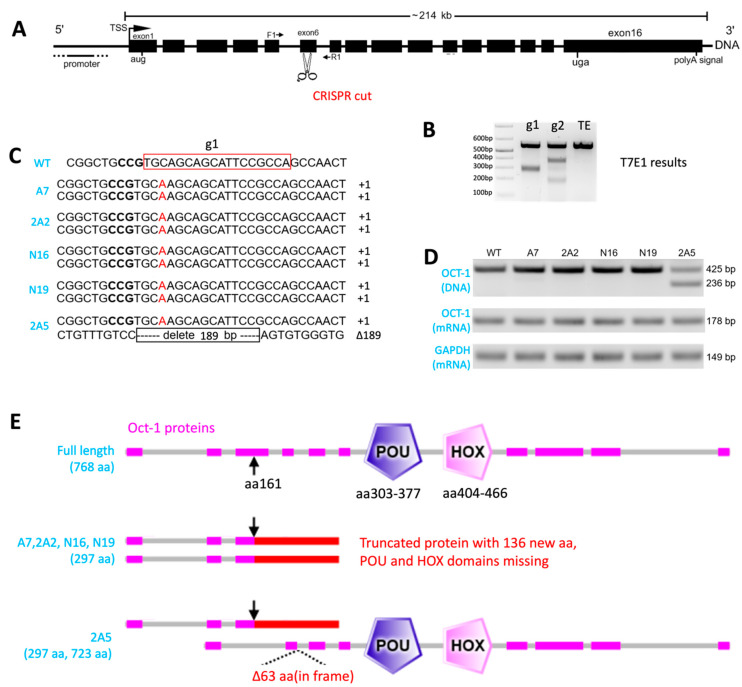
Establishment of Oct1-deficient MDBK single cell clones. (**A**) Gene structure of the bovine Oct1 protein. The scissors indicate where the CRISPR generates its double strand break within exon 6, and the arrows indicate primers used for amplifying the Oct1 gene fragment for genotyping. (**B**) T7 endonuclease I digestion of PCR amplicons using primer set F1 + R1 from cells transfected with sgRNA g1 or g2 to estimate cutting efficiency. (**C**) Genotypes obtained by Sanger sequencing of four homozygous knockout (A7, 2A2, N16 and N19, referred to as Oct1 −/−) and one heterozygous knockout (2A5, referred to as Oct1 −/+) clones used in the study shown. The CRISPR target sequences are denoted by the red rectangle, with the PAM sequence (anti-sense) shown in bold. (**D**) Further genotypic evidence showing the 189 bp deletion in one allele of clone 2A5 (DNA) as well as un-altered Oct1 transcription levels in the edited clones, by reverse transcription and semi quantitative qPCR, relative to the GAPDH housekeeping gene (mRNA). (**E**) Predicted disruption to the open reading frames and Oct1 protein translation in these clones due to the indels.

To examine possible Oct1 involvement during BoHV-1 replication, we generated Oct1-deficient cells using CRISPR/Cas9-mediated genome knockout (KO) [34,35]. To achieve this, we first synthesized two CRISPR sgRNAs, g1 and g2, to target the exon 6 of Oct1 (Figure 1A) by in vitro transcription (IVT), with DNA templates generated by PCRs using the primers specified in Table 1. After electroporating MDBK cells stably expressing Cas9 (Cas9+/+) [28] with these sgRNAs, we conducted a T7 endonuclease I digestion assay and found that both guides induced efficient DNA cutting (Figure 1B). We then performed dilutional cloning using the mixed population transfected with g1 as seeds. By Sanger sequencing, we identified four homozygous single cell clones (−/−, A7, 2A2, N16 and N19) with a single nucleotide “A” insertion in both alleles. We also obtained a heterozygous clone (+/−, 2A5) with the ‘A” insertion in one allele and a 189bp deletion in the other allele, which resulted in a smaller 236 bp band after PCR and electrophoresis (Figure 1C,D).

Our in silico analyses predicted protein loss in these modified clones. Even though semi-quantitative RT-qPCR failed to detect any nonsense mediated decay and reduction at the Oct1 mRNA level (Figure 1D), the 1nt “A” frame disrupting insertion in these clones (A7, 2A2, N16, N19, and 2A5) should lead to protein truncation. It should also result in the loss of the POU and HOX domains (Figure 1E), based on our SMART domain prediction [36] of full-length bovine Oct1 protein (NCBI Ref: XP_005203481). In the 189 bp in-frame deletion allele of 2A5, only 63 amino acid residues were deleted (Figure 1E), leaving the POU and HOX domains unaffected. Unfortunately, due to the lack of a functional Oct1 antibody, we were unable to verify this by Western blotting. Nevertheless, our data provide evidence for the complete loss of the Oct1 protein function in the homozygotes (A7, 2A2, N16, N19, and 2A5) and mono-allelic Oct1 KO in clone 2A5.

### 3.2. Bovine Herpesvirus 1 (BoHV-1) Replication Is Less Efficient in Oct1-Deficient MDBK Cells

**Figure 2:**
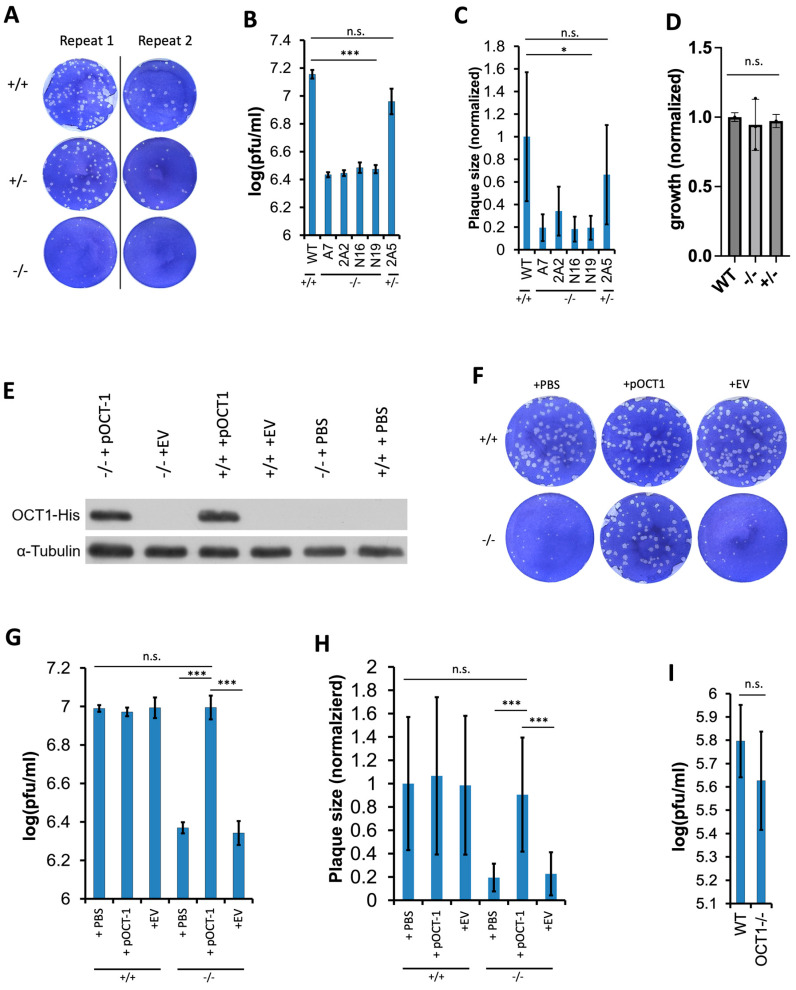
The loss of the Oct1 protein negatively affects replication of BoHV-1, but not that of AIHV-1. (**A**–**C**) Plaquing efficiency in the Oct1 deficient MDBK cells, with representative results shown. Monolayers of the modified cells (−/− and +/−) along with the wild-type were infected with serial diluted BoHV-1 viruses (VP26-GFP) [37]. After four days of incubation, the cells were fixed and stained prior to plaque counting and size measurements (n ≥ 2). Representative plaque assay results are shown (**A**). Plaquing efficiency represented as viral titers in various cell lines (**B**) with sizes of the plaques also measured (**C**). (**D**) Growth of WT, −/−, and +/− cell lines over a 5-day period with each data point normalized to that of the average of WT cells. (**E**–**H**) Complementation of Oct1 in the knockout cells (clone A7) to rescue the impaired BoHV-1 production. Western blot validation of His tagged Oct1 cDNA overexpression (+pOCT1) in the Oct1 −/− cells transfected with a piggyBac vector for stable integration. An empty vector (+EV) or PBS was used instead during electroporation to serve as negative controls, and α-Tubulin was used as a protein loading control. Representative plaque assay results by direct plaquing on cells with or without overexpression of the His tagged Oct1 cDNA (**E**). Plaquing efficiency in the cells with (+pOCT1) or without (+EV or PBS) Oct1 overexpression represented as virus titers (**F**), with the plaque sizes also measured and their averages shown (**G**). (**I**) Plaquing efficiency of AIHV-1 in the Oct1 −/− or WT cells shown after directly infecting monolayers of these cells with serial diluted AIHV-1 cells. All data were expressed as mean ± standard error (SE) and two-tailed Student’s t-tests (n ≥ 2) were performed to compare results between cell lines (n.s., not significant; *: *p* < 0.05; ***: *p* < 0.001).

To find out whether the loss of Oct1 would affect BoHV-1 replication, we then examined BoHV-1 replication in the KO clones. We proceeded with plaquing BoHV-1 directly on these KO clones, and stained the cell monolayer with toluidine blue, a non-viral specific dye that enables counting plaques under an optical microscope. With this method, we found that the plaquing efficiency of BoHV-1 was significantly reduced in the −/− clones compared to wild-type cells (+/+), indicated by fewer and smaller plaques (Figure 2A), as represented by virus titer (Figure 2B) and average diameter of plaques (Figure 2C). We also observed a bigger reduction in virus titer and plaque sizes in the −/− clones relative to the +/− clone 2A5 (Figure 2A–C). Such reductions were unlikely caused by any loss of cell fitness, as we failed to observe any impairment to the growth rate of the KO cells through a 5-day incubation period (Figure 2D). We tried to rescue the reduction by stably express His-tagged bovine Oct1 cDNA in these −/− cells (representative clone A7) via piggyBac transposition (pOCT1, with PBS or empty vector EV as negative controls). After confirming the over-expression by Western blot (Figure 2E), we examined the plaquing efficiency of BoHV-1 in the −/− cells or rescued cells (+pOCT1) like before. We found that Oct1 overexpression in the −/− cells completely restored its plaquing efficiency to the level of the wild-type cells, as measured by plaque numbers presented as virus titers and sizes (Figure 2F–H).

Besides BoHV-1, we also tested the plaquing efficiency of alcelaphine herpesvirus type 1 (AIHV-1) on these cells. AIHV-1 is a gamma herpesvirus which infects wildebeests and cattle [38]. We observed a modest but nonsignificant reduction in the number of plaques formed in the KO cells compared to WT cells (Figure 2I). Taken together, these data suggest that the loss of Oct1 negatively affected BoHV-1 replication, and this reduction was more specific to BoHV-1 rather than AIHV-1. This specificity could be due to the possible lack of strong bovine Oct1 binding sequences in the AIHV-1 genome.

**Figure 3:**
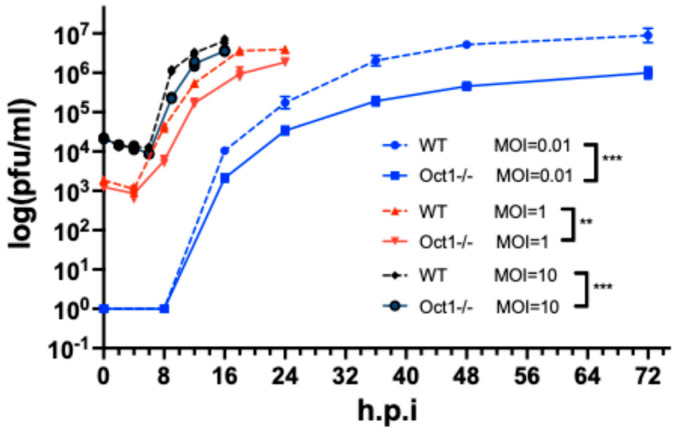
Oct1 knockout cells produce less infectious BoHV-1 viruses than non-edited cells. Growth curves of BoHV-1 in Oct1 −/− and WT cells across 72 h after inoculation at three different MOIs (10, 1, and 0.01). The time-course experiment was performed in triplicate, and every total virus sample was titrated in duplicate on wild-type MDBK cells. The viral titers were plotted with the *y*-axis on a log scale and the geometric means plus 95% CI are displayed for each time point. The significance in differences between the two time series are highlighted (**, *p* < 0.01; ***, *p* < 0.001 with two–tailed Student’s *t*-test, n = 3).

We then conducted time-course experiments to evaluate BoHV-1 viral growth in the Oct1 −/− cells. After infecting the −/− cells (clone A7) and control cells (WT) at different multiplicities of infection (MOI), we collected total virus samples from them at various timepoints across 72 h. We then determined the viral titers of these samples by plaque assays. We observed significant decreases in viruses produced by the −/− cells throughout the course of infection at all three MOIs (0.01, 1 and 10), with the biggest decreases seen in the MOI = 0.01 group (up to 10.5-fold reduction in the MOI = 0.01 group, 6.3-fold reduction in the MOI = 1 group, and 2.6-fold reduction in the MOI = 10 group, Figure 3). There was an inverse relationship between the MOI at infection and reduction in viral titers collected at the end of the infection course: the smallest decrease in virus production was recorded from −/− cells infected at high MOI = 10 infection, and the biggest decrease was recorded with the lowest MOI, at 0.01. These results indicate that the loss of Oct1 leads to less efficient production of infectious BoHV-1 virus. In addition, infecting cells with BoHV-1 at high MOI (MOI = 10) overcame the negative impact of Oct1 loss to some degree.

### 3.3. Oct1 KO Reduces the Expression of All Three Classes of BoHV-1 Genes

So far, our data demonstrate the negative impact Oct1 deficiency has on BoHV-1 replication in MDBK cells, suggesting the importance of Oct1 for BoHV-1. To find how Oct1, a transcription factor, could be involved in BoHV-1 replication, we profiled the expression dynamics of the three gene classes of BoHV-1, immediate early (IE), early (E), and late (L). To achieve this, we first infected Oct1 −/− cells (A7) and the control cells (WT) at either MOI = 10 or 0.1 and cultured the infections for 24 h. We then measured mRNA levels of four IE genes (bICP4, CirC, bICP0, bICP22) and three suspected IE genes (UL21, UL33 and UL34), five E genes (UL5, UL23, bICP27, UL7 and UL8), eight L genes (gD, UL35, gB, UL19, UL46, UL47, UL48 and UL49), and one undefined transcriptional regulated gene (US2) [12,39] by RT-qPCR using total RNA collected at seven time points (0, 4, 8, 12, 16, 20, 24 hpi).

**Figure 4:**
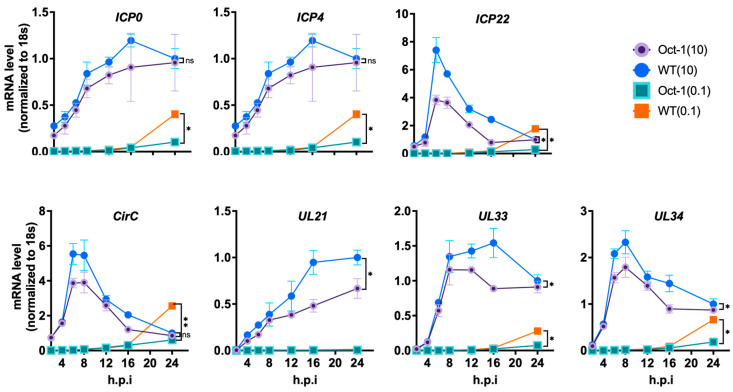
mRNA expression dynamics of the three classic IE genes along with four suspected IE genes in Oct1 −/− and WT MDBKs after BoHV-1 infection. Confident IE genes: ICP0, ICP4, ICP22, and CirC; suspected IE genes: UL21, UL33, and UL34. Monolayers of MDBK cells were infected with the virus at MOI of 0.1 or 10 or mock infected, and total RNAs were extracted from the cells at specified timepoint (from 2 h to 24 hpi). After genomic DNA removal and reverse transcription, relative mRNA levels were quantified by qPCR, using 18S rRNA as a normalizing factor for template input. All calculated values were then normalized to that of WT samples at 24 hpi with an MOI of 10. The data were expressed as mean ± SD and analyzed using multiple unpaired t-tests and the two-stage step-up method in Prism GraphPad (Benjamini, Krieger, and Yekutieli) (ns: *p* > 0.05, *: *p* < 0.05; **: *p* < 0.01, n = 2).

**Figure 5:**
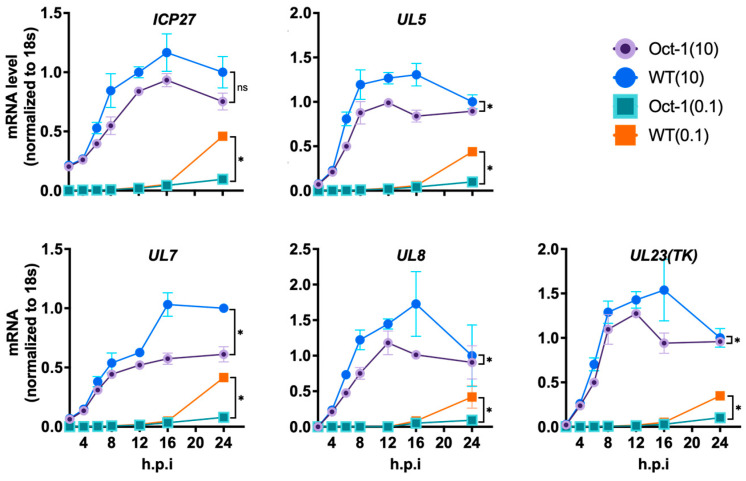
mRNA levels of five E genes of BoHV-1 in Oct1 −/− and WT MDBK cells infected at MOI = 0.1 or 10. The experimental setup, sample collection, processing, and data analyses were all conducted as described in the Figure 4 legend and the Materials and Methods section.

**Figure 6:**
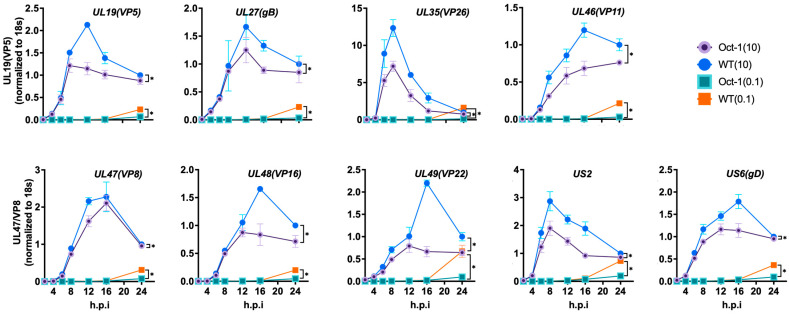
mRNA expression levels of eight L genes of BoHV-1 and one uncategorized gene (US2) in WT or Oct1 −/− cells. The experimental setup, sample collection, processing, and data analyses were all conducted as described in the Figure 4 legend as well as in the Materials and Methods section.

We found that the expression levels of BoHV-1 genes from all three temporal classes were lower in Oct1 KO MDBK cells compared to control cells. Out of the seven IE genes tested, three (ICP22, UL34, UL33) had reduced levels of transcription in the Oct1 KO cells after infection at MOI = 10, while all seven of them exhibited reduced transcription when the cells were infected at MOI = 0.1 (Figure 4). Similar trends with a wider spread of differences were observed for transcriptions of the E (Figure 5) and L (Figure 6) classes of genes; most genes tested were expressed at a significantly lower level in Oct1 −/− cells infected with either MOI = 10 or MOI = 0.1 (all 14 genes tested except ICP27 in cells infected at MOI = 10, Figure 5). Similar results were observed when cells were infected at MOI = 1 (Figures S1–S3). Our RT-qPCR profiling of BoHV-1 gene expression showed that all three temporal classes of BoHV-1 genes were significantly reduced in Oct1 −/− MDBKs cells compared to wild-type cells, indicating that Oct1 facilitates efficient transcription of these genes.

## 4. Discussion

BoHV-1 is an important pathogen for the cattle industry. Better understanding of its interaction with host cell factors is key to developing effective prevention and therapeutic strategies. In this paper, we sought to understand the interaction between BoHV-1 and host transcription factors, and specifically whether Oct1 could play a positive role during viral gene transcription.

To achieve this, we first created Oct1-deficient (bi-allelic or mono-allelic KO) cells by CRISPR/Cas9 gene targeting, utilizing Cas9-expressing MDBK cells as published previously (Figure 1) [28]. By directly plaquing BoHV-1 on these KO cells using our plaque formation assay described in the Materials and Methods section, we found that the plaque formation efficiency was significantly reduced in both the Oct1 −/− and Oct1 +/− MDBKs compared to WT cells, as represented by fewer numbers and smaller sizes of plaques formed (CTRL, Oct1 +/+; Figure 2). Interestingly, this impairment was more pronounced in the Oct1 −/− cells than Oct1 +/− MDBKs (Figure 2A–C), indicating that the infection of BoHV-1 in MDBK cells is Oct1 dosage dependent. This was further supported by our complementation experiment in which over-expressed Oct1 protein completely rescued BoHV-1 production in Oct1 −/− MDBKs (Figure 2E–G). A caveat with our plaque formation assay is that it would miss foci formed by just a few cells or single infected cells. Thus, to better assess the plaquing defect caused by the Oct1 KO, future experimentation using fluorescent detection of viral proteins will be helpful. In addition to plaque formation efficiency, viral growth was also significantly reduced in cells missing Oct1, with an up to 10-fold decrease in virus titers (Figure 3). However, this reduction was modest compared to the three-log differences we observed in our host trafficking complex knockout experiments [28].

We then conducted transcriptional studies to understand the mechanism behind the impaired viral replication. It has been shown that the VP16 protein from either HSV-1 or BoHV-1 can recruit the human Oct1 protein to form a transcriptional regulatory complex [40]. Further studies have demonstrated that Oct1 is a critical factor for efficient IE gene expression during HSV-1 replication [21,41]. This led us to investigate whether bovine Oct1 would be important for BoHV-1 gene expression. By reverse transcription and qPCR quantification of viral RNA, we showed that all three classes of BoHV-1 gene expressions were affected by Oct1 knockout (Figure 4, Figure 5 and Figure 6), with bigger differences observed in cells infected at low MOI (MOI = 0.1) compared to high MOI (MOI = 10). This is consistent with results obtained using HSV-1 infection, which demonstrated that Oct1 was only critical in low MOI but not high MOI infections [21].

These data suggest that viral infections at high MOI overcome the impairment, possibly due to functional similarity and redundancy between Oct1 and other POU domain family members. The redundancy is also indicated by the modest reduction in virus titers produced by these KO cells (Figure 3). Another explanation could be that the viral tegument proteins, such as ICP4, which is present in the BoHV-1 virion [37] and carried across the cell membranes by the input virions at high MOI infections, are sufficient to kickstart viral transcription. Taken together, our findings serve as direct evidence to confirm that the bovine Oct1 protein acts as a pro-viral transcription factor for BoHV-1 infection, like human Oct1 to HSV-1, even though it has been shown that the two VP16 proteins from BoHV-1 and HSV-1 induce differential changes in Oct1 conformation upon binding [22,23].

For this study we conducted our experiments in MDBK cells. Unfortunately, because the MDBK cells were of epithelial origin, these cells and our infection model were not ideal for the study of neurotrophic infection and latency. It has been suggested that Oct1 levels are downregulated in neuronal cells upon HSV-1 infection [42], and the failure to form a transactivation complex consisting of VP16, Oct1, and HCF-1 in neurons facilitates the establishment of latency [43]. In addition to downregulation of Oct1, competitive binding of VP16 by other cellular factors such as Luman [44] and Zhangfei [45] might also contribute to the establishment and maintenance of latency. It will be interesting to further investigate the role that Oct1 and other cellular factors play during BoHV-1 latency, if a BoHV-1 latency model using bovine neuronal cells can be obtained, e.g., via programmed stem cell differentiation.

## Figures and Tables

**Table 1:** Primers used for sgRNA synthesis by in vitro transcription and for genotyping KO cells.

Primers	Primer Sequences (5′–3′)
T7-g1 For.	TTAATACGACTCACTATAG*G**CTGGCGGAATGCTGCTGCA***GTTTAAGAGCTATGCTGG
T7-g2 For.	TTAATACGACTCACTATAGG***TCTGTATGGGCTGAGACAAG***GTTTAAGAGCTATGCTGG
IVT rev. primer	AAAAGCACCGACTCGGTGCC
F1 for. primer	TGCCATATGAAGTTGGGTAGC
R1 rev. primer	CCTCTGCTCCAGAAATACGC

Note: the DNA sequence in bold is the CRISPR used for the KO.

## Data Availability

All data generated in this study have been included in this manuscript.

## Supplementary Materials

The following supporting information can be downloaded at: https://www.mdpi.com/article/10.3390/v16101549/s1, Figure S1: Expression of seven immediate-early genes assayed by qRT-PCR in WT or Oct1 KO MDBK cells infected at MOI = 1. Figure S2. Expression levels of five early genes of BoHV-1 in Oct1 −/− and WT MDBK cells infected with MOI = 1, at the mRNA level assayed by qRT-PCR. Figure S3. Expression of six late genes of BoHV-1 after infecting WT or Oct1 −/− cells at MOI = 1 assayed by qRT-PCR.; Table S1. Primers used to amplify Oct1 from cDNA and cloning into a piggyBac vector. Table S2. primers used in semi-quantitative RT-PCR for assaying Oct1-KO MDBK cells. Table S3. Primer used for detecting BoHV-1 genes and host gene 18S by RT-qPCR.
