# Supplementary material for: Bovine Transcription Factor POU Class 2 Homeobox 1 (POU2F1/Oct1) Protein Promotes BoHV-1 Replication in MDBK Cells
Source: Viruses. 2024 Sep 30;16(10):1549. doi: 10.3390/v16101549 (PMC11512350; doi:10.3390/v16101549)
Supplement: Supplementary file 1 [file viruses-16-01549-s001.zip › viruses-3120687-supplementary.pdf]

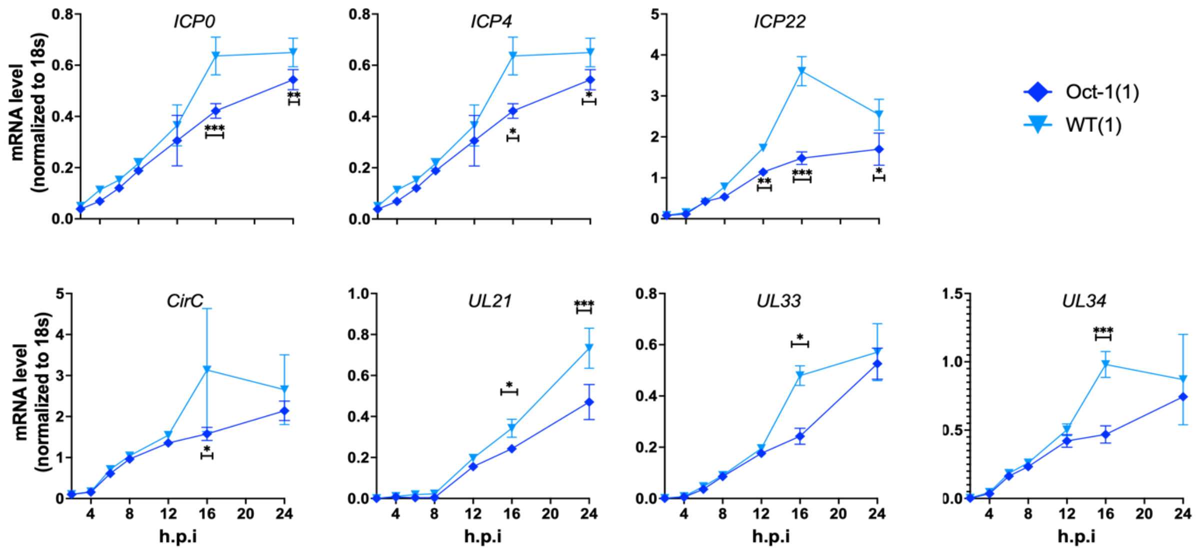

Figure S1. Expression of seven immediate-early genes assayed by qRT-PCR in wt or Oct1 KO MDBKcells infected at MOI = 1. This figure is supplementary to Figure 4 in the main text.

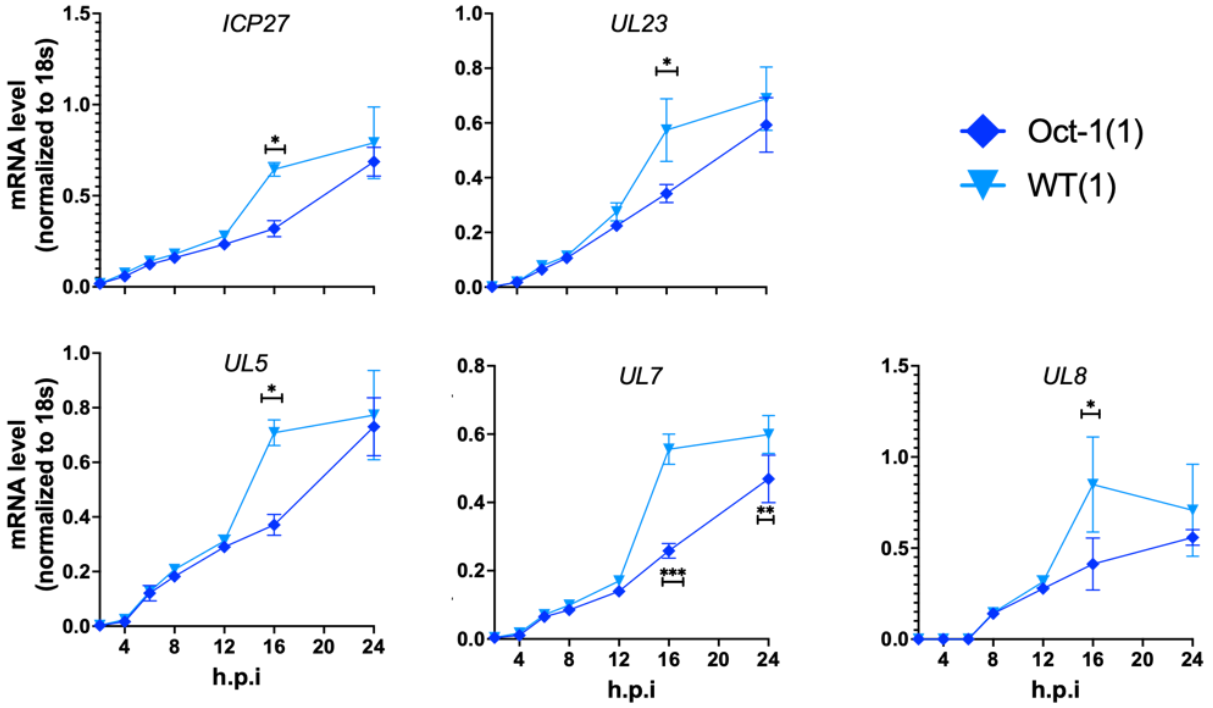

Figure S2. Expression levels of five early genes of BoHV-1 in Oct1$^{-/-}$ and wt MDBK cells infected with MOI=1, at the mRNA level assayed by qRT-PCR. This figure is supplementary to Figure 5 in the main text.

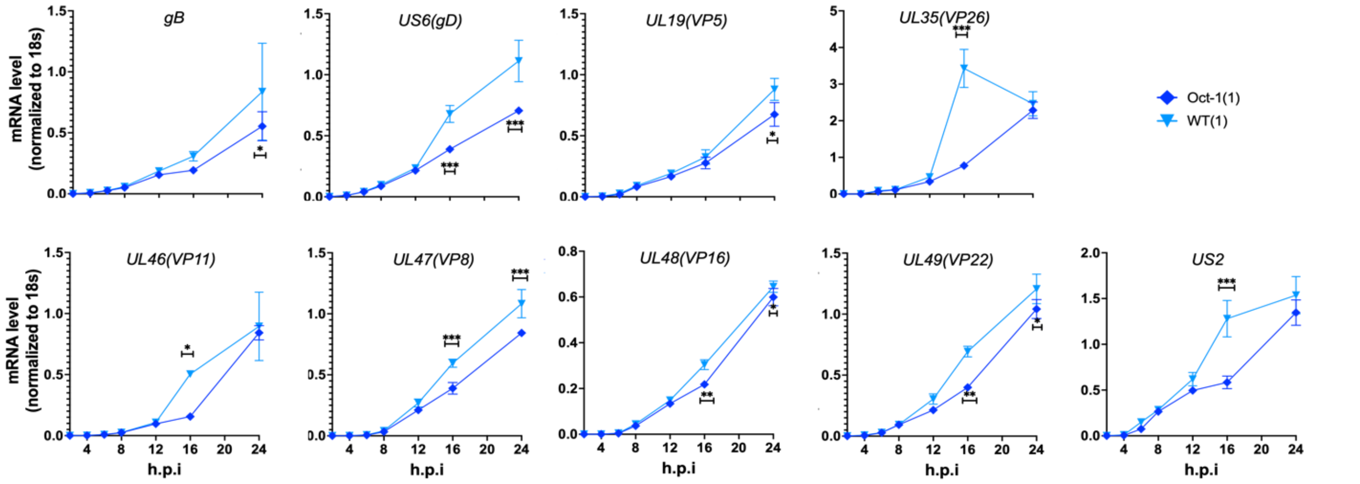

Figure S3. Expression of six late genes of BoHV-1 after infecting wt or Oct1 -/- cells at MOI=1 assayed by qRT-PCR. This figure is supplementary to Figure 6 in the main text.

Table S1. Primers used to amplify Oct1 from cDNA and cloning into a piggyBac vector.

Gene	Primer sequences (5′-3′)
OCT1	CATTTTGGCAAAGAATTCCCTAGG*ACGCGT***GCCACCATG**GCGGACGGAGGAG-CAGCGAGTC<br>TCTACAAATGTGG-TATTCGAA*GTTTAAAC*TCA**ATGGTGGTGGTGATGATG**CTGCGCCTT-GGAGGCGGTGGTGGTG

Table S2. primers used in semi-quantitative RT-PCR for assaying Oct1-KO MDBK cells.

Primers	Primer sequences (5′-3′)	Length
OCT1-2	CTTCAGCCAAACTACCATCTCTCGT<br>GTTCAAGCCCTCAATCCCCAT	178 bp
GAPDH	CTGACCTGCCGCCTGGAGAAA<br>GTAGAAGAGTGAGTGTCGCTGTT	149 bp

Table S3. Primer used for detecting BoHV-1 genes and host gene 18S by RT-qPCR.

Gene	Primer sequences (5′-3′)	Gene	Primer sequences (5′-3′)
18S	TGTGATGCCCTTAGATGTCC<br>TTATGACCCGCACTTACTGG	*UL35*	GCAGATTTTGCATGTGCTAAACGCC<br>CGTGGTCGTAGGTCGCAAACAT
bICP4	CGGAGAGCAGCGAGGACGACGG<br>GCTTCGATGGCGGCGGCTATGA	*UL34*	CGGCCAGGACGGAAGCAACGAG<br>CGCGGGCCTTGATCTTCTCCAGC
bICP0	GCTGTGGATGAGAGACTGCACGG<br>TGGGATCGTGCTGCATCTGC	*UL33*	CCCCCCGAGGCGCTGGC<br>CCCGCCCGAGCCGTGTGC
bICP22	TCGCCATCGACGTCAACACCCT<br>CGTGTAGCCGAGCAGGAAGAAG	*UL21*	GGGGGCGCGTTTGTGGACTG<br>GCCCGAGAGCGCGTTGGTGAT
gD	CGAGCCCAGGAAGCACTTTG<br>ACCGTGCCGTCGATGTACAG	*UL5*	CGTTTACTGCTGGTGGCTCATCAAT<br>TGGTGATAAGGCAGGTGAGGACATT

U_L46	TGCCGCTTCGCTCACTTTAC	U_L8	GACTACTGCTTCTTGCTGGGGTTTC
	TCAGCAGCGCCAGCTTAAAC		AATGCGGCGGTTCAGGCTTGC
U_L47	TGCCCAGCCTCAGGTAGAG	U_L23	CTCTGCTACCCCTTCGCCCGCTACT
	GTGGTTATTAGGAGCCCATGC		AGGGTGCACACGACGAGGTTGGC
U_L48	GATCAACTACGGGACGACTCCA	*gB*	CACGGACCTGGTGGACAAGAAG
	CGCCTGAGCCAGATGTGTG		CTACCGTCACGTGCTGTGTAC
U_L49	CCTACAACCGAACGATCTTTTGCGA	*bICP27*	GCTGTTGGAGGTGACTGTGGGC
	ACTTCAACATGCGATCCAATCGCTC		TACACGGGAACCAGGGTGAAAT
U_L7	CGACGTTCACGTCATCGTCCATTAC	*CirC*	CCCTGCCGCAAGTTTATGCTGTAT
	TACGCATCGCAGTCCACTTCTTCG		GTAAGAAAGTCGTGCAGTGAATCGG
U_L19	ACGAGTGGCTGGTGCTGGCTAA	*US2*	TAGTAAAAACGGCGAAGGGCTGC
	ATGTTGGTCACGGCTTCCTGC		CCCGAGTTCAGCAAGTTGTAGCC
